# Removal of electrostatic artifacts in magnetic force microscopy by controlled magnetization of the tip: application to superparamagnetic nanoparticles

**DOI:** 10.1038/srep26293

**Published:** 2016-05-19

**Authors:** Livia Angeloni, Daniele Passeri, Melania Reggente, Diego Mantovani, Marco Rossi

**Affiliations:** 1 Department of Basic and Applied Sciences for Engineering, SAPIENZA University of Rome, Via A. Scarpa 16, 00161 Rome, Italy; 2 Lab. for Biomaterials and Bioengineering (CRC-I), Dept. Min-Met-Materials Eng. & University Hospital Research Center, Laval University, Quebec City, Canada; 3 Research Center for Nanotechnology applied to Engineering of SAPIENZA University of Rome (CNIS), Piazzale A. Moro 5, 00185 Rome, Italy

## Abstract

Magnetic force microscopy (MFM) has been demonstrated as valuable technique for the characterization of magnetic nanomaterials. To be analyzed by MFM techniques, nanomaterials are generally deposited on flat substrates, resulting in an additional contrast in MFM images due to unavoidable heterogeneous electrostatic tip-sample interactions, which cannot be easily distinguished from the magnetic one. In order to correctly interpret MFM data, a method to remove the electrostatic contributions from MFM images is needed. In this work, we propose a new MFM technique, called controlled magnetization MFM (CM-MFM), based on the *in situ* control of the probe magnetization state, which allows the evaluation and the elimination of electrostatic contribution in MFM images. The effectiveness of the technique is demonstrated through a challenging case study, i.e., the analysis of superparamagnetic nanoparticles in absence of applied external magnetic field. Our CM-MFM technique allowed us to acquire magnetic images depurated of the electrostatic contributions, which revealed that the magnetic field generated by the tip is sufficient to completely orient the superparamagnetic nanoparticles and that the magnetic tip-sample interaction is describable through simple models once the electrostatic artifacts are removed.

The increasing interest in the study and the development of magnetic nanomaterials for different technological applications^1^,^2^,^3^ has highlighted the need of new tools and procedures for the characterization of magnetic properties at the nanometer scale. Conventional techniques, such as superconducting quantum interference devices (SQUID) magnetometry^4^ or vibrating sample magnetometry (VSM)^5^, are widely used for the characterization of magnetic nanomaterials, but the magnetic characterization of single nanomaterials can be achieved only through the use of techniques which combine the capability of positioning and imaging at the nanometer scale with the probing of ultra-low magnetic fields. Accurate mapping of ultra-low magnetic field distribution has been demonstrated through the use of scanning magnetometry with Nitrogen-vacancy (N-V) color centers in diamond^6^,^7^,^8^,^9^, which however requires a quite complex experimental setup. Therefore, despite the growing interest in these methods, the use of techniques based on simpler setups is still more widespread. Among them, magnetic force microscopy (MFM) is considered a promising technique thanks to its lateral resolution comparable to transmission techniques (10–20 nm), its applicability to all kinds of nanomaterials without any particular sample preparation, both in air and in liquid, its high magnetic sensitivity and its capability to map the magnetic evolution of a sample with respect to an applied field^10^,^11^,^12^,^13^. Nevertheless, despite the wide employment of MFM technique for the qualitative characterization of magnetic nanomaterials, only a few studies have been performed using MFM for the measurements of their magnetic properties^14^,^15^,^16^,^17^,^18^. The difficulty of obtaining reliable results is ascribable to a certain inconsistency between the experimental data and the theoretical models describing the magnetic tip-sample interactions. This incongruence has been mainly attributed to the not satisfactory description of the probe, generally assumed as a single magnetic point dipole^19^,^20^, and several attempts have been made to find more accurate mathematical approaches^21^. Such proposed models generally take into account only the magnetic tip-sample interactions, but different authors^22^,^23^ recently demonstrated that the signal detected by MFM contains also a significant contribution due to long-range electrostatic phenomena, which include the effect of fixed electric charges on the sample as well as of topography-modulated tip-sample capacitive coupling. Therefore, it should be more realistically described as the sum of a magnetic and an electrostatic contribution. Consequently, the evaluation of the effects of electrostatic forces appears essential to obtain accurate magnetic measurements by MFM. Only a few studies have been carried out with the aim of distinguishing or eliminating the electrostatic signal in MFM images and a few methodologies have been proposed. For example, in the case of homogenous samples, the electrostatic contribution can be eliminated compensating the tip-sample contact potential difference by the application of an appropriate bias voltage^24^. Nevertheless, if the analyzed sample is heterogeneous as in the case of magnetic nanomaterials deposited on flat substrates, the contact potential difference depends on the actual position of the probe on the sample surface and the electrostatic contribution cannot be removed by the application of a single, fixed, bias voltage value. In order to evaluate and eliminate the electrostatic contribution also in the case of heterogeneous samples, Jafaar *et al*.^22^ proposed the combined use of the Kelvin probe force microscopy (KPFM) and MFM techniques, the former allowing the measurement of the contact potential difference in each point of the scanned area and its compensation by opportunely adjusting the applied bias voltage during the scan. In switching magnetization MFM (SM-MFM), proposed by Cambel *et al*.^25^,^26^, the analyzed surface is scanned twice in tapping mode, with opposite tip magnetization orientations, obtained by applying an opportune magnetic field before each scan. If the magnetization state of the sample is not affected by the external field applied to invert the probe magnetic moment and by the magnetic field induced by the tip during the measurements, reversing the probe magnetization results in the inversion of the detected magnetic contrast while the atomic and electrostatic contributions remain unchanged. Thus, adding the traces obtained with opposite tip moments the magnetic signal is annulled and only the contrast due to the atomic and electrostatic tip-sample interactions is visible. On the contrary, subtracting the same two traces the electrostatic signal is nullified and only the magnetic tip-sample interactions give rise to the contrast in the image. Differential MFM is an analogous method recently proposed by Wang *et al*.^27^, in which the two MFM images with reversed polarization are acquired subsequently to the topography with the tip maintained at a fixed distance (lift height) from the surface (lift mode). The applicability of these techniques for the evaluation of the electrostatic and magnetic signal is limited to hard ferromagnetic materials, having significant remanent magnetic moment and coercivity sufficiently high to ensure a constant magnetization of the sample even after the application of the external magnetic field necessary to invert the probe magnetization and under the magnetic stray field induced by the tip during the scan. Due to this limitation, SM-MFM and differential MFM are not applicable to the most of magnetic nanomaterials, which exhibit magnetic characteristics near to the superparamagnetic limit (i.e., having low or zero coercivity).

In this work, we propose a new MFM technique, which we refer to controlled magnetization MFM (CM-MFM), which allows the evaluation and the elimination of electrostatic contribution in MFM images by controlling the tip magnetization state. The effectiveness of the technique is verified through its application to the investigation of superparamagnetic nanoparticles (NPs) in absence of external field, which represents one of the most challenging targets of MFM. Magnetic images were acquired and rationalized describing both the tip and the NP as magnetic dipoles, demonstrating the suitability of this simple model when MFM data are depurated of the electrostatic contributions.

## Magnetic force microscopy

Magnetic force microscopy (MFM) is a particular scanning probe microscopy (SPM) technique, which allows one to detect tip-sample magnetostatic interaction forces and to image them on the sample surface. This is obtained using an atomic force microscopy (AFM) setup equipped with a magnetic tip, generally consisting in a standard AFM Si probe coated with a magnetic layer with thickness of a few tens of nanometers. The cantilever is set into oscillation at a frequency *f* close to its first free resonance frequency *f*_0_. When the probe is brought sufficiently close to the sample surface, the gradient along the *z* axis of the tip-sample force *F* produces a variation in the dynamic behavior of the cantilever, which can be described as a change in the phase shift:




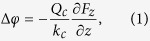




where *Q*_*c*_ and *k*_*c*_ are the cantilever quality factor and spring constant in air and with the tip not interacting with the sample, respectively, and 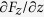 is the gradient along the vertical axis *z* of the vertical component of the (magnetostatic) tip-sample interaction force (*F*_*z*_)^28^. The actual expression of *F*_*z*_ depends on both the geometry and the magnetic domains configuration of both the tip and the sample. For example, on the basis of experimental data the former has been modeled using either a single point magnetic dipole, a cone with uniformly magnetized magnetic surface, or more exotic magnetic structures^21^,^29^,^30^. Also, different analytical expression for *F*_*z*_ are obtained in case the sample is a single magnetic dipole or a more complex structure with periodic magnetic domains^29^,^31^,^32^. In particular, let us suppose that the sample is represented by a single small magnetic NP with diameter *d* uniformly magnetized with magnetization *M*_*s*_, possibly coated with a nonmagnetic layer with thickness *c*_*s*_, which we describe as a single point magnetic dipole with magnetic momentum *m*_*s*_ = 1/6*πM*_*s*_*d*^3^. If the tip can be modeled by a punctiform magnetic dipole with momentum *m*_*tip*_, the magnetic phase shift Δ*φ* observed when the tip is placed on the top of the NP, i.e., the symmetry axes of the tip and the NP coincide, can be described using a one dimensional analytical model which is justified by the symmetry of the problem. In this case, Δ*φ* is given by




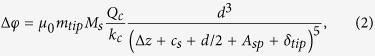




where *μ*_0_ is the permeability of free space, Δ*z* is the lift height (the vertical distance between the tip apex and sample surface, i.e., the top of the NP), *A*_*sp*_ is the set-point amplitude during the first pass in tapping mode, and *δ*_*tip*_ is the position of the equivalent momentum *m*_*tip*_ evaluated from the tip apex^29^,^33^. MFM images are generally acquired in the so-called ‘lift height mode’. In this two-pass modality, each line is scanned twice and two different images of the selected sample area are recorded. The first scan is performed in standard tapping mode to acquire and record the topography, while the second scan is performed in non-contact mode, in order to detect only long-range interaction forces (e.g., magnetic and electric forces) and obtain a magnetic map of the sample. During this second scan, the probe follows the trajectory of the previously recorded sample profile at a selected distance Δ*z* (the lift height), which is maintained constant at each point (*x, y*) of the scanned area, in order to eliminate any possible artifact in the magnetic signal due to the variation in the long-range the tip-sample interaction forces produced by the modulation of the actual tip-sample distance.

## Controlled magnetization MFM

The experimental setup to perform controlled magnetization MFM (CM-MFM) consists in a standard MFM apparatus equipped with an electromagnet placed under the sample, which allows one to apply controllable out-of-plane static magnetic fields *H* in the range −480 Oe < *H* < +480 Oe to the tip-sample system, without moving the probe from the scan area. Similar systems have been already applied to vary the magnetization state of the sample in order to study its magnetic evolution in response to magnetic fields^34^,^35^,^36^,^37^,^38^. Conversely, here the system is used to *in situ* control the magnetization state of the probe. The measurement procedure consists in two different phases: (i) the calibration of the remanent magnetic behavior of the MFM tip and (ii) the measurement of the ‘only magnetic’ MFM contrast by recording and opportunely post-processing two MFM images of the same sample area, acquired with two different magnetization states of the probe.

## Step I: Probe calibration

The calibration phase consists in the individuation of the characteristic parameters of the remanent hysteresis loop of the probe. This hysteresis curve is the plot, as a function of the value of magnetic field applied and then switched off, of the remanence corresponding to the in-field minor hysteresis loop where the maximum magnetization corresponds to the actual value of the magnetic field applied and then switched off^39^,^40^,^41^,^42^. The parameters to be determined to calibrate the tip are the remanent saturation magnetic field *H*_*rs*,*tip*_ and the remanent coercivity *H*_*rc*,*tip*_. Different calibration methods have already been developed for the characterization of the in-field magnetic characteristics of the MFM probes^43^,^44^,^45^. Here, we propose a simple method to measure the remanent hysteresis loop of the MFM probe. The procedure consists in the measurement of the MFM contrast detected on a high coercivity reference sample after the application and the subsequent switching-off of out-of-plane magnetic fields with different intensities. We use a commercial floppy disk as a reference sample. The out-of-plane coercivity of this kind of samples, significantly higher than the probe coercivity, allows us to ascribe the changes in the MFM contrast exclusively to the variation of the magnetization state of the sensor. At the same time, the well defined magnetic structure, consisting in a periodic pattern of in-plane domains alternatively oriented in opposite direction, allows us to easily measure the variations of the phase contrast in response to the changes in the magnetization state of the tip. When the tip magnetization is directed perpendicularly to the sample surface, the MFM contrast is maximum in correspondence of the domains transitions (positive or negative depending on the mutual direction of the involved tip and sample magnetic domains), where the magnetic field generated by the floppy has only vertical component^32^. Conversely, the MFM contrast is zero in correspondence of the internal domains regions, where the magnetic field generated by the floppy has only horizontal component^32^. Therefore, the (out-of-plane) remanent hysteresis loop of the probe can be obtained plotting the MFM phase difference Δ*φ*^*cont*^ between two adjacent transition regions (i.e., the image contrast) as a function of the previously applied (and eventually switched-off) external magnetic field *H*. Δ*φ*^*cont*^(*H*) is a function of the remanent magnetization of the tip and the sample (*M*_*r*,*tip*_ and *M*_*r*,*sample*_, respectively). *M*_*r*,*tip*_ depends on the applied magnetic field *H*. Conversely, if a sample with high out-of-plane coercivity like the floppy disk is used, *M*_*r*,*sample*_ is independent of *H*.

Thus, the normalized remanent hysteresis curve of the MFM probe can be obtained




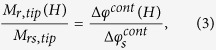




where 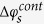 is the MFM phase difference between two adjacent transition regions detected when the remanent magnetization of the tip reaches its saturation value (*M*_*rs*,*tip*_).

**Figure 1:**
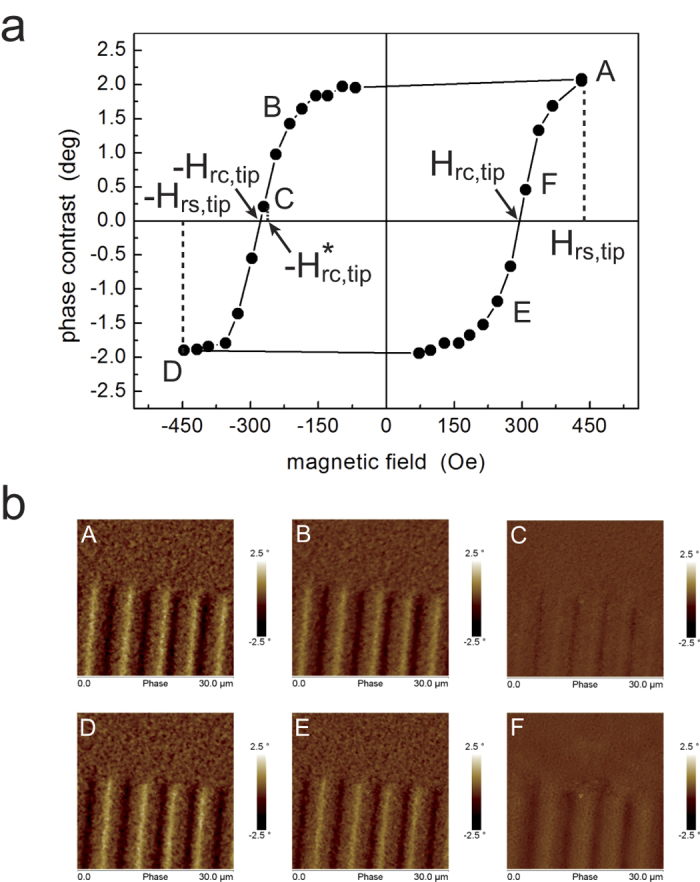
Experimental characterization of the remanent magnetic properties of a standard MFM tip. (**a**) Hysteresis curve of the MFM phase contrast as a function of the magnetic field applied and subsequently switched off. (**b**) Examples of MFM images of the periodic magnetic domains of a standard floppy disk, from which the phase difference between two adjacent transition regions was measured in order to determine the points of the hysteresis curve. Points from A to F in panel (**a**) are obtained from images from A to F in panel (**b**).

A typical remanent hysteresis loop of a standard MFM tip (MESP, Bruker Inc.) measured with this method is reported in Fig. 1a. Experiments were performed using a standard AFM apparatus (Icon, Bruker Inc.) provided with standard MFM imaging technique and equipped with an in-house made CM-MFM setup. The latter is an electromagnet constituted by a coil (with inner diameter 1 cm, outer diameter 2.7 cm, and height 1.6 cm) supplied with direct electric current through a dc power supply and placed under the sample holder. The control of the power supply is external to and independent of the AFM electronics. Therefore, our technique can be implemented on every AFM apparatus, providing that enough room is available for the coil. At the beginning of the experiment, a magnetic field *H* = 480 Oe was applied. Switching off the magnetic field results in a partial demagnetization of the tip, which reaches a near-saturation state corresponding to its maximum remanent magnetization, i.e., its remanent saturation. An image of the magnetic domains of the floppy was acquired (image A in Fig. 1b, from with the value of Δ*φ*^*cont*^ marked with A in Fig. 1a is determined). Then, several MFM images were recorded after the application and the switching off of magnetic fields in the opposite direction with increasing intensities (e.g., images B and C in Fig. 1b corresponding to the point B and C in Fig. 1a) down to a magnetic field *H* = −480 Oe at which the saturation of Δ*φ*^*cont*^ in the opposite direction is observed (image D in Fig. 1b and point D in Fig. 1a). Then, positive values of *H* are applied to complete the hysteresis curve (e.g., images and points E and F). From the curve reported in Fig. 1a, it is possible to individuate both the saturation magnetic field *H*_*rs*,*tip*_ necessary to obtain the saturation remanent magnetization of the probe, corresponding to its maximum magnetic sensitivity, and the coercive magnetic field *H*_*rc*,*tip*_ necessary to annul the remanent magnetization of the probe^46^.

## Step II: Determination of the magnetic signal

Once the remanent properties of the probe have been determined, the magnetic moment of the tip can be *in situ* controlled through the application and the subsequent switching off of appropriate magnetic fields. In particular, +*H*_*rs*,*tip*_ must be applied to magnetize the tip, while −*H*_*rc*,*tip*_ or −*H*_*rs*,*tip*_ must be applied to annul or invert the tip magnetization, respectively.

**Figure 2:**
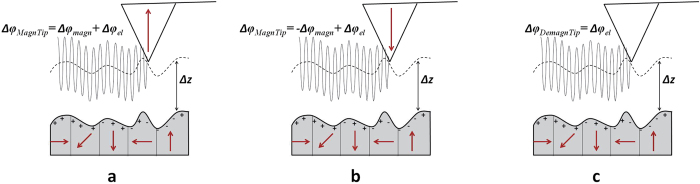
Sketch of the tip-sample interactions in CM-MFM. The sample is assumed constituted by magnetic domains with different orientation (red arrows) and by distributed electric charges which are responsible for a tip-sample electrostatic interaction not uniform on the surface. The three configurations of the tip are characterized by different magnetization of the tip: (**a**) tip with saturated ‘up’ magnetization; (**b**) tip with saturated ‘down’ magnetization; (**c**) demagnetized tip.

In the CM-MFM procedure, a first scan of the area is performed at fixed Δ*z* with the tip magnetized in its saturation state (having applied and then switched off a magnetic field +*H*_*rs*,*tip*_) and a ‘standard MFM image’ is acquired. As previously discussed, such an image is affected by both magnetic and electrostatic tip-sample interactions. Indeed, the ‘standard MFM signal’ obtained with the magnetized tip (Δ*φ*_*MagnTip*_) is actually the superimposition of both the ‘true’ magnetic signal (Δ*φ*_*mag*_) and the electrostatic signal (Δ*φ*_*el*_), i.e.,




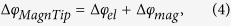




which is schematically represented in Fig. 2a. After the first scan, a magnetic field with intensity −*H*_*rc*,*tip*_ is applied and switched off. A second image is acquired with the same instrumental parameters and the same lift height Δ*z*, but with the probe having zero magnetization (Fig. 2c). In this case, the signal detected with the demagnetized tip Δ*φ*_*DemagnTip*_ is represented by the only electrostatic contribution, i.e.,




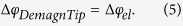




Therefore, the magnetic contribution, which in the following we refer to as the CM-MFM signal Δ*φ*_*CM*–*MFM*_, can be obtained by subtracting the second image to the first one, i.e.,




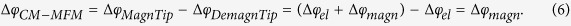




The use of this mode, which we called *zero probe magnetization* (ZM) mode, allows the detection of the electrostatic and magnetic tip-sample interactions independently of the magnetization state of the sample, enabling the analysis of soft ferromagnetic, paramagnetic and superparamagnetic materials, to which the SM-MFM and differential MFM are not applicable. The same CM-MFM instrumentation can be also used to distinguish the electrostatic and magnetic signals in MFM images of relatively hard ferromagnetic samples, the stray field of which could orient the domains of the tip, thus reducing the effectiveness of the tip demagnetization procedure. Indeed, a magnetic field with intensity −*H*_*rs*,*tip*_ can be applied and switched off after the first scan, following a procedure analogous to that used in SM-MFM^25^,^26^ or in differential MFM^27^. A second MFM image is recorded with the probe magnetized along the opposite direction with respect to the first one (Fig. 2b) and the electrostatic and magnetic contributions can be evaluated by adding or subtracting the two images, respectively.

**Figure 3:**
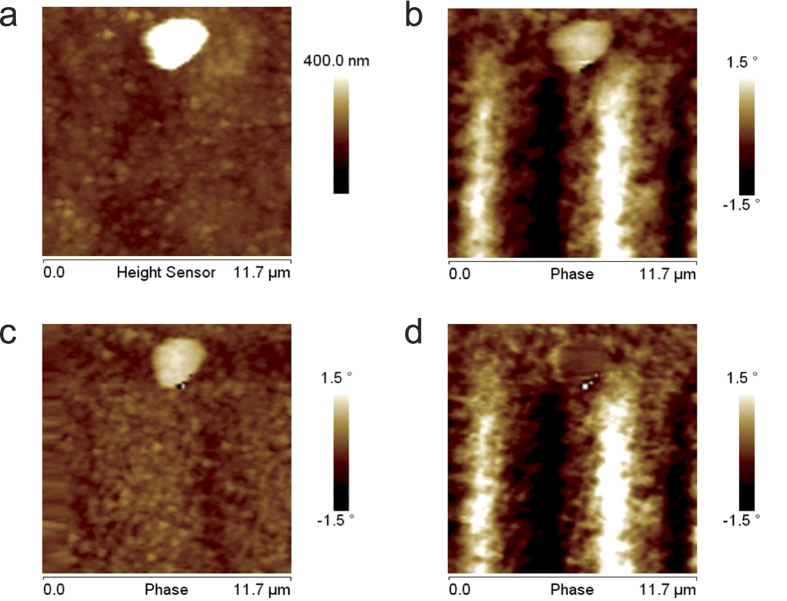
CM-MFM characterization of a standard floppy disk. (**a**) Topography of an area where a particle (likely dust) is observable on the floppy surface and (**b**) corresponding standard MFM phase image acquired with the magnetized tip. (**c**) Phase image acquired with the demagnetized tip. (**d**) CM-MFM phase image obtained by subtracting (**c**) from (**b**).

An example of application demonstrating the effectiveness of the method is shown in Fig. 3, in which the characterization of a standard floppy disk using CM-MFM in ZM mode is reported. The topography (Fig. 3a) shows the presence of a particle (likely dust) with height of some hundreds of nanometers. The corresponding bright contrast (Δ*φ* = +1.42 deg) in standard MFM image (Fig. 3b) would suggest a repulsive tip-sample interaction, which however can hardly find a convincing physical rationalization. The same contrast Δ*φ* = +1.42 deg is observed in the electrostatic image acquired with the demagnetized tip (Fig. 3c), compatible with the presence of an electrostatic interaction produced by the tip-sample capacitive coupling^23^. After subtraction, the magnetic image is obtained (Fig. 3d), where the magnetic domains of the floppy are correctly visualized but no contrast (Δ*φ* = 0 deg) is observed in correspondence of the particle. This result experimentally demonstrates the potential capability of our method to compensate electrostatic phase shift signal resulting from the tip-sample capacitive coupling in magnetic images.

The value of *H*_*rc*,*tip*_ can be easily determined with enough accuracy from the curve in Fig. 1a as the intersection with the horizontal axis of the linear curve fitting of the two points immediately below and above it. From a conceptual point of view, such a value can always be applied to completely demagnetize the MFM probe. The sensitivity of the dc power source used to generate the magnetic field, however, could prevent the application of the exact value of *H*_*rc*,*tip*_, as it is discussed in details below. It is worth explicitly discussing the range of possible samples that can be investigated using CM-MFM. As detailed above, CM-MFM in ZM mode is particularly suitable for the analysis of superparamagnetic NPs, which cannot be investigated by SM-MFM and differential MFM as the NP magnetization is reversed together with that of the tip, preventing the inversion of contrast in the two magnetic images. The technique is also effective on relatively hard magnetic samples, like the floppy disk, the magnetic domains of which are not affected by the magnetic fields applied during the magnetization and demagnetization procedures and which generate magnetic fields not sufficient to (even partially) polarize the demagnetized tip. In particular, the latter issue was explicitly verified in the case of floppy disk. If the magnetic field it generates could orient the demagnetized tip, no alternation between dark and bright stripes in correspondence of domain transitions would be observed in the phase shift images, resulting in a pattern of only dark (attractive) stripes with halved spatial period. Apart from these experimental evidences, the weakness of the field generated by the floppy is confirmed by its rough estimation from the phase contrast values it produces and using analytical models present in literature^32^. We calculated this field to be lower than 10^−2^ Oe at a lift height of 100 nm and thus negligible with the respect to *H*_*rc*,*tip*_. The magnetization of the tip by the magnetic stray field of the sample, which has never been observed with the cantilevers we used in our experiments but it is likely to occur with low-coercive tips, limits the application of our technique on magnetic materials much harder that the floppy. In principle, these materials could be analyzed selecting different tip with higher coercivity. The magnetic field generated by these materials, however, would be so intense that electrostatic artifacts would be negligible. Really, even in the case of the standard floppy disk, the topography induced electrostatic artifacts modulate the standard MFM response but they are negligible with respect to the magnetic signal, so that the latter is well described by analytical models which consider only magnetic tip-sample interactions^32^. Also, it should be observed that MFM mainly targets to nanomaterials, which are unlikely to generate such intense magnetic fields, being more realistically near to the superparamagnetic limit. Conversely, materials with magnetic properties similar to those of the tip cannot still be analyzed with our technique. Indeed, even if the magnetic field they produce cannot polarize the demagnetized tip, the demagnetization/magnetization procedure could modify the orientation of their magnetic domains. This would not represent a limit if in-field measurement are required, but prevents the possibility of investigating their ‘pristine’ magnetic state.

## Case study: analysis of superparamagnetic nanoparticles

The effectiveness and the potentialities of the CM-MFM approach have been demonstrated through the study of superparamagnetic NPs, which probably represent the most challenging kind of sample for the sensitivity of standard MFM. Indeed, their nanometer size comparable with the MFM lateral resolution and their low magnetic moments make the magnetic tip-sample forces comparable with the corresponding electrostatic interactions^23^. Furthermore, because of their superparamagnetic character, if the MFM measurements are carried without applying any external magnetic field, the magnetization of the NPs is only due to the magnetic field induced by the probe during the scan and, thus, is always oriented along the same direction of the probe magnetization. The SM mode is thus inapplicable, while the ZM mode of CM-MFM can be used to decouple electrostatic artifacts from MFM images.

**Figure 4:**
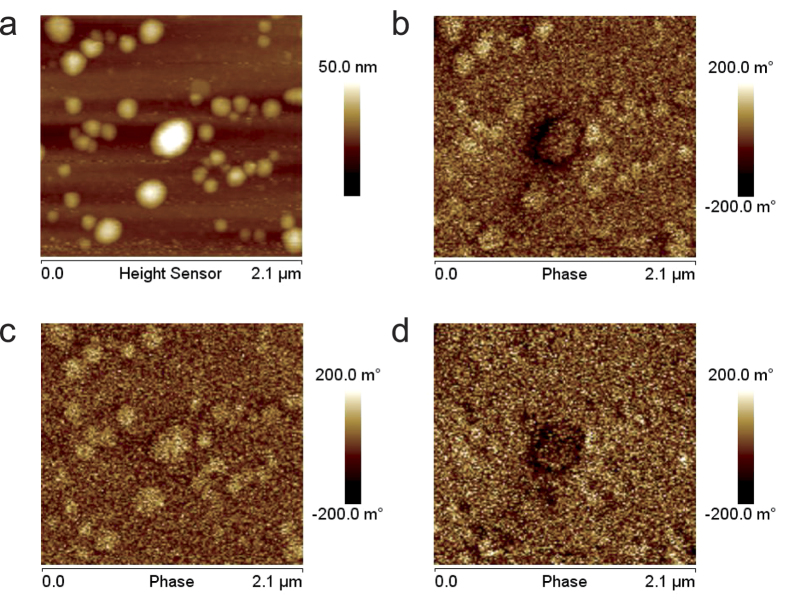
CM-MFM characterization of superparamagnetic NPs. (**a**) Topography of an area where some NPs are visible and (**b**) corresponding standard MFM phase image acquired with the magnetized tip. (**c**) Phase image acquired with the demagnetized tip. (**d**) CM-MFM phase image obtained by subtracting (**c**) from (**b**).

The test sample was prepared depositing a colloidal solution of commercial Fe_3_O_4_ NPs (Sigma Aldrich) with nominal diameter of 20 nm on a clean monocrystalline Si substrate. The analysis was carried out using a MFM tip with a standard momentum (MESP, Bruker Inc.). During the measurement session, both topographic and MFM images of the investigated areas were continuously acquired. Subsequent images of the same area could allow us to monitor possible gradual demagnetization of the tip as well as the occurrence of abrupt phenomena, e.g., destructive tip-sample contacts or snatching of NPs, which however have never occurred during the whole experiment. The calibration of the tip was performed following the previously illustrated procedure, revealing a remanent saturation field *H*_*rs*,*tip*_ = 440 Oe at which 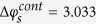 deg is measured. At the beginning of the experiment, the probe was magnetized through the application of a magnetic field with intensity *H*_*rs*,*tip*_. The magnetic field was switched off and ‘standard MFM’ images of NPs were recorded, the sample being magnetized only through the magnetic field induced by the tip. Then, a magnetic field −*H*_*rc*,*tip*_ was applied (and switched off) in order to annul the tip magnetization. Electrostatic images of the same sample area were then recorded with the demagnetized probe. CM-MFM images were obtained by subtracting the electrostatic images to the standard MFM images. An example of this CM-MFM characterization in ZM mode is reported in Fig. 4. In particular, Fig. 4a,b show the topography and the standard MFM image acquired at Δ*z* = 20 nm, respectively, of an area of the sample where the biggest NP with diameter *d* = 30 nm is surrounded by smaller NPs with diameters *d* < 20 nm. In the image recorded with the magnetized tip (Fig. 4b) a slight negative contrast was detected in correspondence of the biggest NP, indicating a weak attractive tip-NP interaction. Conversely, a positive contrast was detected in correspondence of smaller NPs, indicating a repulsive tip-sample interaction. Because of their superparamagnetic character, in absence of an external magnetic field, NPs can be magnetized only by the tip magnetic stray field and, thus, only an attractive magnetic tip-sample interaction can occur. Consequently, the observed positive contrast cannot be attributable to magnetic phenomena. This result indicates the presence of a significant electrostatic contribution in standard MFM images, which results to be even higher than the magnetic one in correspondence of small NPs. In the phase image acquired with the demagnetized tip (Fig. 4c) a positive contrast is observed in correspondence of all the NPs, i.e., also in the bigger one which exhibited a negative contrast in the images acquired with the magnetized tip. This confirms the presence of a significant positive electrostatic contribution in correspondence of all the analyzed NPs. Although the positive phase shift in correspondence of the NPs may indicate a difference between their electric properties and those of the substrate, it is more likely attributable to a topography-induced artifact due to the capacitive tip-sample coupling, i.e., to the reduction of the tip-sample attractive forces in correspondence of the NPs produced by the increasing of the average tip-sample distance^23^. Finally, Fig. 4d shows the image resulting form the subtraction of Fig. 4c (electrostatic contribution detected with the demagnetized tip) to Fig. 4b (standard MFM image acquired with the magnetized tip which is affected by both the electrostatic and the magnetic contribution). It represents the magnetic contribution, depurated of the electrostatic effects. A larger negative contrast is observable in correspondence of the NP which exhibits a negative contrast in standard MFM images (*d* = 30 nm). Conversely, only a slight negative contrast is observed in correspondence of small NPs (*d* < 20 nm) which can be hardly distinguished from the noise. This can be ascribed to the small volume of the NPs and, thus, to their magnetic stray field, lower than the probe sensitivity.

**Figure 5:**
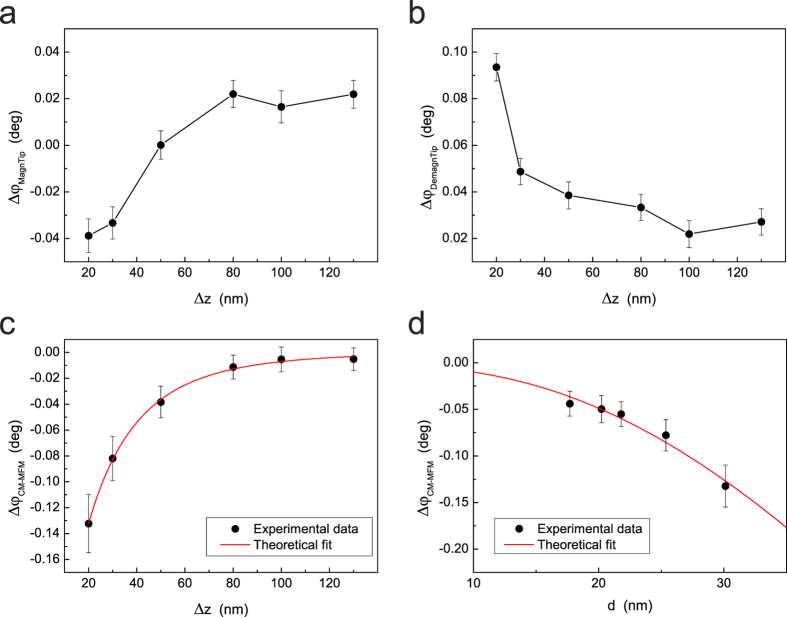
Analysis of images collected using CM-MFM technique in ZM mode. (**a**) Standard MFM phase contrast (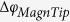) as a function of the lift height (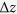) measured on a NP with diameter *d* = 30 nm using a magnetized probe, which is affected by both electrostatic and magnetic tip-sample interactions. (**b**) Phase contrast on the same NP as a function of the lift height acquired with the demagnetized tip (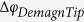), which is affected only by the electrostatic tip-sample interactions. (**c**) CM-MFM phase contrast (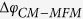) as a function of the lift height obtained by subtracting data in (**b**) from those in (**a**) with the corresponding fit using the simple model of two magnetic dipoles in Eq. (2). (**d**) CM-MFM phase contrast (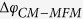) as a function of the NP diameter obtained analyzing five NPs with the corresponding fit using the simple model of two magnetic dipoles in Eq. (2).

In order to further confirm the magnetic nature of the tip-sample interaction detected in CM-MFM, we carried out an analysis of the phase contrast as a function of the tip-sample distance (i.e., lift height Δ*z*) and of the NPs diameter *d*. Several images of the same sample area with different Δ*z* have been recorded with both the magnetized and the demagnetized probe. For each image, the absolute value of the phase shift detected in correspondence of the NPs has been determined as the difference between the mean values of the phase measured inside the NP and in correspondence of an adjacent region of substrate. The uncertainty in the measured values has been determined combining the statistics on a small area corresponding to the top of the NP and on the selected region of substrate. Figure 5a shows an example of standard MFM phase contrast Δ*φ*_*MagnTip*_ as a function of the lift height Δ*z* obtained with the magnetized probe on a NP with *d* = 30 nm. In correspondence of small tip-sample separations (Δ*z* < 50 nm), an attractive interaction with the NP is experienced by the tip (negative phase shift), which can be ascribed to the predominance of magnetic interactions in this region. Increasing the tip sample distance, the repulsive force decreases until becoming null and then attractive (Δ*z* > 50 nm), indicating the predominance of electrostatic forces at large tip-sample separations. This is congruent with the faster distance-decay of magnetic forces (expected to be proportional to *z*^−4^) with respect to the electrostatic ones (expected to be proportional to *z*^−2^)^47^. Nevertheless, although the general trend of the data is not difficult to justify, the actual Δ*φ*_*MagnTip*_(Δ*z*) data reported in Fig. 5a can be hardly rationalized through simple analytical models and their not monotonic behavior is apparently ascribable only to the occasional presence of a random bias in different MFM images. An analogous Δ*φ*_*DemagnTip*_(Δ*z*) curve on the same NP obtained with the demagnetized probe is reported in Fig. 5b, which represents the electrostatic signal. As expected, it is always positive, indicating a reduced tip-sample attractive force, and decreases with the increasing of the distance. Also in this case, data can be hardly rationalized through simple analytical models. Nevertheless, by subtracting the data acquired with the demagnetized probe (Fig. 5b) to those acquired with the magnetized probe (Fig. 5a) the CM-MFM signal Δ*φ*_*CM*–*MFM*_ is obtained (Fig. 5c). It turned out that a negative phase shift indicating an attractive (i.e., magnetic) tip-NP interaction exists also at distances larger than 50 nm. This was not detectable in standard MFM images because of the preponderance, at large tip-sample distances, of the electrostatic contribution with respect to the magnetic one. Thus, in absence of any external magnetic field, the NP is magnetized by the tip stray field also when the probe is at distance larger than 50 nm. This produces an attractive magnetic interaction which, nevertheless, is not detectable by standard MFM measurements due to the predominant electrostatic contribution, but which can be revealed using CM-MFM in ZM mode. It is interesting to notice that while both the standard MFM data (Fig. 5a) and the electrostatic ones (Fig. 5b) cannot be described by simple models and seem affected by a remarkable uncertainty, the magnetic data (Fig. 5c) show a monotonic trend. Moreover, the fit of Δ*φ*_*CM*–*MFM*_(Δ*z*) data reported in Fig. 5c using Eq. (2) demonstrates that they are very well described by the simple model in which both the tip and the NP are represented with punctiform magnetic dipoles. In particular, being performed the images with set-point amplitude *A*_*sp*_ = 28 nm, from the fit an experimental value of *δ*_*tip*_ = 39 ± 4 nm is determined. This result indicates that, when the MFM signal is depurated of the effect of electrostatic tip-sample forces, a MFM tip acts as a single-point dipole. By averaging the results of an analogous analysis of data obtained on three NPs with different diameters, a value of *δ*_*tip*_ = 45 ± 7 nm has been obtained. Fig. 5d reports the Δ*φ*_*CM*–*MFM*_(*d*) data measured at Δ*z* = 20 nm together with the corresponding fit which has been obtained using Eq. (2) assuming *δ*_*tip*_ = 45 nm. Also in this case, the experimental curve is well described by Eq. (2) although the relatively narrow distribution of NP diameters does not allow us to present more efficacious results. Considering that in these experimental conditions the minimum value of phase shift we can reveal is 10^−2^ deg, we can evaluate that with the present settings and equipment our technique could allow the study of NPs with diameter not smaller than 10 nm. Interestingly, our results demonstrate that the weak magnetic field generated by the tip is sufficient to completely orient the magnetic domains of superparamagnetic NPs, which is a debated issue in the scientific community of MFM users^29^,^48^. Moreover, as a result of the removal of electrostatic signal in MFM images through CM-MFM, the tip-sample interaction is found to be describable with the simple one dimensional model of two interacting magnetic dipoles.

## Current limits and future perspectives

The results reported in this work demonstrate that, in principle, CM-MFM may represent a powerful technique to delete electrostatic artifacts resulting from tip-sample capacitive coupling in MFM images. Thus, CM-MFM images can be used to deduce information on local magnetic properties of materials, e.g., magnetic momentum or magnetization, with nanometer lateral resolution. Despite the potentiality and the correctness of its working principle, however, we must point out some current limitations of our technique, mainly due to practical issues related to the experimental setup which basically lead to the incomplete demagnetization of the probe. Understanding and solving these limitations represent the main challenges of our current work of improvement of CM-MFM.

Obviously, being a two-pass technique, the correctness of topographic images is an essential prerequisite for the accuracy of CM-MFM. Artifacts in the reconstruction of the topography, e.g., due to incorrect choice of instrumental parameters like set-point, scan rate, or feedback gain, result in artifacts in the magnetic images which cannot be corrected. This problem is somewhat more severe in CM-MFM as two subsequent images of the same area have to be acquired, with the magnetized and the demagnetized tip, respectively.

These issues affect CM-MFM as well as any other two-pass technique. In addition, since the MFM phase shift depends on the instrumental parameters (e.g., drive frequency and amplitude, set-point amplitude), the same parameters must be used in the calibration on the reference sample (e.g., the floppy) and in the analysis of the investigated sample (e.g., the NPs).

Other severe limitations can occur due to the incomplete demagnetization of the tip. Depending on the sensitivity of the power supply, indeed, the experimental setup is characterized by a minimum step allowed between two values of the applied magnetic field. In our case, for instance, the minimum allowed step between two values of magnetic field is 15 Oe. Apart from particular and occasional cases in which −*H*_*rc*,*tip*_ coincides with one of applicable values of magnetic field, this demonstrates that with our experimental setup we cannot reach the complete demagnetized state of the tip. Indeed, referring to Fig. 1a, we determine −*H*_*rc*,*tip*_ = −278.0 ± 7.5 Oe (which is in agreement with the (in-field) coercivity of about 400 Oe reported by the manufacturer) while the closest value of magnetic field we can apply is 
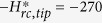
 Oe. In correspondence of 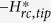 we observe a contrast in the images equal to 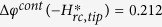 deg, which is 10% of the saturation contrast 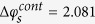 deg. As Δ*φ*^*cont*^ is proportional to the tip magnetization, the signal detected with the (partially) demagnetized tip includes not only the electrostatic contribution but also a part of the magnetic one. Therefore, when subtracting the two signals obtained with the magnetized and (partially) demagnetized tip, respectively, the signal reflecting the magnetic tip-sample interaction is also modified. The extent of this effect can be evaluated, and the effect itself can be corrected, in the simple case of a sample the magnetization of which is independent from that of the tip. In this case, Eq. (5) can be rewritten as




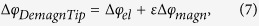




where *ε* is a demagnetization ratio given by 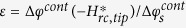. Notably, the value of *ε* can be determined from the MFM contrast on the floppy since on such a sample not only are electrostatic effects negligible with respect to the magnetic ones, but also because they are uniform over the surface. Therefore they are deleted when subtracting the maximum and minimum phase shifts in the MFM images to evaluate the phase contrast^32^. In this case, instead of by Eq. (6), the CM-MFM signal is given by




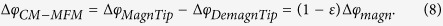




Thus, despite the not complete demagnetization of the tip, the electrostatic contribution is completely deleted in Δ*φ*_*CM*–*MFM*_, but the magnetic signal is underestimated or overestimated. This effect can be corrected since the magnetic signal can be obtained as




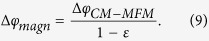




**Figure 6:**
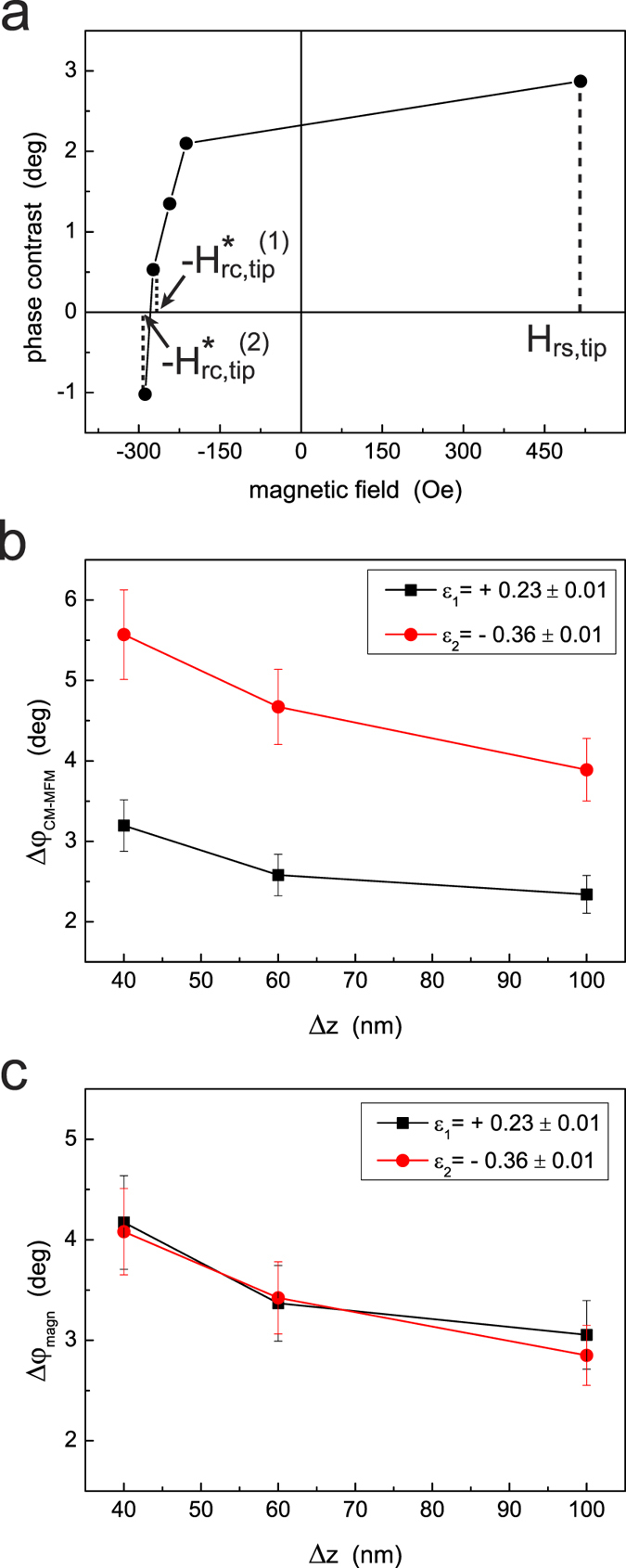
(**a**) Tip calibration on the floppy, from which the remanent saturation magnetic field *H*_*rs*,*tip*_ and two values near the remanent coercive field are determined. (**b**) CM-MFM signal (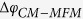) as a function of the lift height (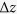) measured on the floppy in two cases of nearly demagnetized tip, characterized by two values of the demagnetization factor *ε*. (**c**) Corrected magnetic signals (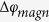) as a function of the lift height (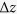), obtained using Eq. (9).

As *ε* can be easily determined from the MFM images of the floppy reference sample, our technique allows us to obtain the correct intensity of the magnetic signal depurated from the electrostatic contribution when the sample magnetization is independent of that of the probe. It is worth noting that the same result can be obtained even if 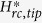 is not chosen as close as possible to *H*_*rc*,*tip*_ providing the corresponding value of *ε* is determined. Minimizing the residual magnetization of the tip (and thus *ε*), however, ensures the highest signal-to-noise ratio. The consistency of this correction method is demonstrated by data reported in Fig. 6. From the calibration of the probe on the floppy (Fig. 6a), the value of *H*_*rs*,*tip*_ = 510 Oe is obtained at which the remanent magnetization of the tip can be considered saturated. Two values of magnetic field are determined at which the tip can be considered nearly demagnetized, i.e., −
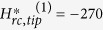
 Oe and −
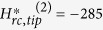
 Oe, respectively. These correspond to not null phase contrast values equal to 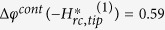 deg and 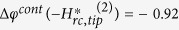 deg (note that the the contrast is reversed in correspondence of −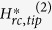), respectively. The values of phase contrast were determined from statistics on lines corresponding to maximum and minimum of the phase of the floppy domains in a certain area. Being 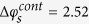 deg, in correspondence of these two nearly demagnetized states of the tip the values *ε*_1_ = 0.23 ± 0.01 and *ε*_2_ = −0.36 ± 0.01 are determined, respectively, where the negative sign of *ε*_2_ is due to the reversal of the contrast in correspondence of −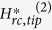. In a different area of the sample, the contrast between two selected points was used to determine the Δ*φ*_*CM–MFM*_ signal as a function of the lift height Δ*z* in the two cases of nearly demagnetized tip. As clearly shown in Fig. 6b, although Δ*φ*_*CM–MFM*_ is supposed to represent the ‘magnetic only’ signal, two completely different curves are obtained. This is due to the fact that in the case characterized by *ε*_1_ a fraction of the magnetic signal is actually subtracted and thus the latter is underestimated. Conversely, in the case characterized by *ε*_2_ a fraction of the magnetic signal is added (due to reversal in sign of the phase contrast) and thus the latter is overestimated. After correction using Eq. (9), however, the same values of Δ*φ*_*magn*_ are obtained (Fig. 5c), which confirms the consistency of the method. The correction of CM-MFM data can effectively compensate the incomplete demagnetization of the tip in case of samples the magnetization of which does not depend on that of the tip, and can be applied not only to data obtained on selected points but on the whole CM-MFM images.

If the spin of the sample depends on that of the tip, like in superparamagnetic NPs, the incorrectly subtracted fraction of Δ*φ*_*magn*_ depends also on the magnetization states of the NP when the tip is saturated and nearly demagnetized. For instance, if the magnetic momentum of the NP is saturated neither when the tip is saturated nor nearly demagnetized, the magnetization of the NP is proportional to the magnetic field generated by the tip. This in turns is proportional to the tip magnetization. Therefore, Eq. (7) can be rewritten as




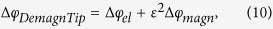




and thus the CM-MFM signal is given by




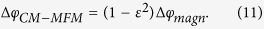




**Figure 7:**
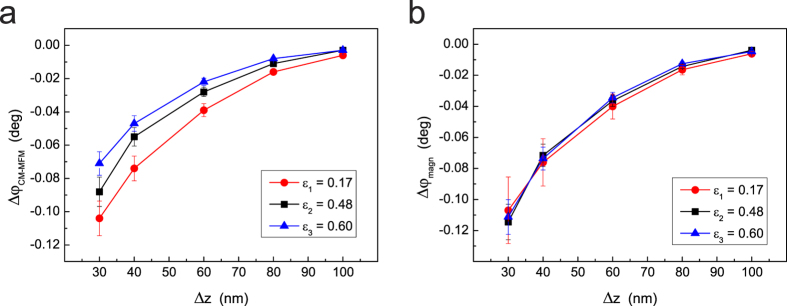
(**a**) CM-MFM signal (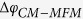) as a function of the lift height (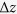) measured on a NP with diameter *d* = 25 nm in three cases of nearly demagnetized tip, characterized by three values of the demagnetization factor *ε*. (**b**) Corrected magnetic signals (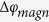) as a function of the lift height (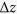), obtained using Eq. (11).

Thus, dividing Δ*φ*_*CM*–*MFM*_ by (1−*ε*^2^), the corrected Δ*φ*_*magn*_ versus Δ*z* curves can be obtained. Depending on the field generated by the tip, however, the magnetization of the NP can be saturated by the magnetized tip but not by the nearly demagnetized one. Moreover, depending on the residual magnetization of the tip, the magnetization state of the NP varies with the lift height. In this case, the ratio between the spin of the NP when the tip is fully magnetized and demagnetized is no longer proportional to *ε*. Thus, the correction factor cannot be easily estimated. In order to demonstrate the effectiveness of this correction procedure in the case of the analyzed NPs, we acquired three sets of CM-MFM phase versus lift height curves using three different values of the demagnetization coefficient, i.e., *ε*_1_ = 0.17, *ε*_2_ = 0.48, and *ε*_3_ = 0.60, determined on the reference floppy as previously described. In Fig. 7a, three of these curves, obtained on a NP with diameter *d* = 25 nm, are reported. The curves clearly show that the bigger *ε* the lower the Δ*φ*_*CM*–*MFM*_ signal, which is obviously due to the bigger fraction of the subtracted magnetic signal a result of a bigger residual magnetization of the (nearly) demagnetized tip. Assuming that the magnetic stray field generated by the saturated tip is not much bigger than the saturation field of the NP, the CM-MFM signal can be corrected and the magnetic signal can be obtained as 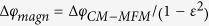. Thus, the curves shown in Fig. 7b are obtained, which demonstrate that the corrected 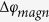 values retrieved using the three different values of *ε* coincide within the experimental uncertainty. Although potentially capable to correct CM-MFM data, the described procedures are admittedly a bit intricate and lengthen the whole CM-MFM procedure introducing additional sources of uncertainty. Also, the sample may contain different types of magnetic nanomaterials and the magnetic field generated by the tip may not uniformly affect them. In this case the correction procedure cannot be applied to the whole image but only to data collected in selected points. Also, even at fixed lift height the correction factor may be not constant over the sample surface. In addition, we note that the uncertainty in the value of the applied magnetic field results in an uncertainty in the values of the correction factors (which is included in the error bars in the graphics in Figs 6c and 7b). As the demagnetization is performed only at the beginning of the experiment and, thus, all the points in the curves are obtained with the very same value of *ε*, its uncertainty acts as a coefficient which multiplies all the points and therefore it must be considered when comparing curves obtained in different measurement sessions, i.e., after different demagnetization steps. Thus, not with standing the possibility of correcting CM-MFM data, the incomplete demagnetization of the tip represents a serious limitation to the accuracy of the technique. In order to overcome this limitation, if one wants to use the approach described in this work, an improved setup should be considered. Enhanced sensitivity of the power supply can ensure a smaller residual magnetization of the tip in its (nearly) demagnetized state. Also, a more effective demagnetization procedure could be selected, e.g., through the use of damped oscillating magnetic fields.

Another current limit of CM-MFM is that the tip calibration procedure for the determination of the coercive field is performed using the floppy reference sample at a certain distance along the axis of the coil. The field generated by the coil, nevertheless, decreases as the distance from the coil along its axis increases. Therefore, if the sample is placed on the top of the coil, variations in the sample thickness result in variations in the distance between the tip and the coil and, thus, in the height at which the tip demagnetization procedure is performed. On samples much thinner or thicker than the floppy (including possible additional substrates), the demagnetization step is performed at height from the coil different from that at which calibration was performed. This leads to an incorrect demagnetization during the experiment with a residual magnetization of the probe significantly bigger than that estimated, dramatically affecting the accuracy of the measurement. In this work, great attention was paid to perform the tip demagnetization at the same height in both the calibration step on the floppy and in the analysis of the NPs. This strategy, however, may be hardly applicable in some specific cases, e.g., if the sample to be analyzed is particularly thick. In this case, no correction can be carried out. A solution to this limitation could consist in the *ex situ* demagnetization of the tip, using a ‘demagnetization station’. Although modern AFM setups ensure a relatively accurate positioning even after macroscopic displacements of tip and sample (e.g., to shift between ‘sample load’ and ‘sample scan’ positions), this procedure may lead to drifts in the imaged area. This may result in more time-consuming experimental session especially when small areas and high resolutions are required. Another solution could be the use of a different demagnetization procedure, i.e., using oscillating damped magnetic field. Depending on the initial values of magnetic field, this procedure could allow a certain margin of variation of the tip-coil distance without compromising the accuracy of the tip demagnetization. Finally, another solution could be the use of a coil integral with the AFM head and, thus, with the tip. This would allow one to perform the demagnetization procedure at fixed and thus correct coil-tip distance. This however could not be implemented on every AFM setup due to the volume and weight of the coil. In particular, it could be hardly used in ‘tip scan’ AFM systems.

Not with standing all the aforementioned limitations, CM-MFM has however the potential, which we have not fully explored yet, for accurate characterization of magnetic parameters of isolated nanomaterials. For example, the curves reported in Fig. 7b can be fitted using Eq. (2) to roughly estimate the mass magnetization saturation of the analyzed NP. Indeed: *k*_*c*_ = 5 N/m and *Q*_*c*_ = 170 have been determined for the used cantilever; *m*_*tip*_ = 1 × 10^−13^ EMU can be assumed, although actually only its order of magnitude is indicated by the producer and thus we cannot be more accurate without its independent measurement; the values of the other parameters can be determined by the fit itself, even if the absence of proper statistics dramatically affects the accuracy of these values. With all these assumptions, *M*_*s*_ = 20 EMU/g is determined which, considering the uncertainty in *m*_*tip*_, is compatible with but smaller than the values (about 60–70 EMU/g) obtained on macroscopic Fe_3_O_4_ NPs samples with similar diameters measured at room temperature by conventional techniques reported in literature^49^. Indeed, we must also add to the previously indicated sources of uncertainty the assumption that the NP is perfectly spherical and that its magnetization is uniform in the volume ignoring possible near-surface effects.

## Conclusions

In conclusion, we have addressed the issue of the effect of electrostatic tip-sample interactions in MFM, which generally prevent the acquisition of magnetic images and thus limit the accuracy of magnetic measurements at the nanometer scale. We developed a MFM-based approach in which the two subsequent images of the same area are collected, one with the magnetized and one with the quasi-demagnetized probe, which is possible after the determination of the remanent saturation and remanent coercivity magnetic fields of the actually used probe performed using a reference sample with periodically patterned magnetic domains.

The effectiveness of our technique is demonstrated through a challenging case study, i.e., the characterization of superparamagnetic NPs in absence of any applied external magnetic field. Images reflecting the magnetic properties of the sample have been obtained subtracting images acquired with the demagnetized tip to the standard MFM images, demonstrating the effectiveness of our techniques in the removal of electrostatic artifacts in MFM maps. Moreover, in addition to the demonstration of the technique, the analysis of our data demonstrated that the magnetic field produced by the magnetized tip is sufficient to completely orient the magnetic domains of superparamagnetic NPs even in absence of any applied external magnetic field. Once the electrostatic artifacts are removed, the tip-sample interaction is well described by that of two single-point magnetic dipoles. The need for an accurate control of the instrumental parameters, the effect of the sample thickness, and the incomplete demagnetization of the probe still represent serious limitations to CM-MFM which must be overcome to increase the accuracy of the technique. Overall, our controlled magnetization MFM technique has been demonstrated to allow us to perform real magnetic characterizations at the nanometer scale.

## Additional Information

**How to cite this article**: Angeloni, L. *et al*. Removal of electrostatic artifacts in magnetic force microscopy by controlled magnetization of the tip: application to superparamagnetic nanoparticles. *Sci. Rep.* **6**, 26293; doi: 10.1038/srep26293 (2016).
